# Utilization of preventive services in a systemic lupus erythematosus population-based cohort: a Lupus Midwest Network (LUMEN) study

**DOI:** 10.1186/s13075-022-02878-8

**Published:** 2022-09-01

**Authors:** Baptiste Chevet, Gabriel Figueroa-Parra, Jeffrey X. Yang, Mehmet Hocaoglu, Shirley-Ann Osei-Onomah, Cassondra A. Hulshizer, Tina M. Gunderson, Divi Cornec, Kamil E. Barbour, Kurt J. Greenlund, Cynthia S. Crowson, Alí Duarte-García

**Affiliations:** 1 Division of Rheumatology, Mayo Clinic, Rochester, 200 First Street SW, MN 55905 USA; 2 Division of Rheumatology, Brest Teaching Hospital; LBAI, UMR1227, Univ Brest, Inserm, CHU de Brest, Brest, France; 3 Department of Internal Medicine, Mayo Clinic, Rochester, MN USA; 4 Department of Quantitative Health Sciences, Mayo Clinic, Rochester, MN USA; 5 Division of Population Health, National Center for Chronic Disease Prevention and Health Promotion, Centers for Disease Control and Prevention, Atlanta, GA USA; 6 Robert D. and Patricia E. Kern Center for the Science of Health Care Delivery, Mayo Clinic, Rochester, MN USA

**Keywords:** Systemic lupus erythematosus, Preventive services, Cancer screening, Osteoporosis, Vaccine, Cardiovascular risk, Diabetes, DXA, Influenza, Zoster, Pneumococcal

## Abstract

**Background:**

Systemic lupus erythematosus (SLE) is a disease that can lead to damage of multiple organs and, along with certain treatments, increase the risk of developing cancer, cardiovascular disease, diabetes, osteoporosis, and infections. Preventive services are particularly important in patients with SLE to mitigate the aforementioned risks. We aimed to evaluate the trends of preventive services utilization in patients with systemic lupus erythematosus, compared with non-SLE population.

**Methods:**

All ≥19-year-old patients in the Lupus Midwest Network (LUMEN) registry, a population-based cohort, with SLE on January 1, 2015, were included and matched (1:1) by sex, age, race, and county to non-SLE comparators. Among both groups, we compared the rates of screenings for breast and cervical cancer, hypertension, hyperlipidemia, diabetes mellitus, and osteoporosis as well as immunizations.

**Results:**

We included 440 SLE patients and 430 non-SLE comparators. The probability of breast cancer screening among women with SLE was similar to comparators (hazard ratio [HR] 1.09, 95% CI 0.85–1.39), while cervical cancer screening was lower (HR 0.75, 95% CI 0.58–0.96). Hypertension screening was higher among patients with SLE (HR 1.35, 95% CI 1.13–1.62); however, hyperlipidemia screening was similar to comparators (HR 1.16, 95% CI 0.96–1.41). Diabetes and osteoporosis screenings were more likely to be performed for SLE patients than for comparators (HR 2.46, 95% CI 2.11–2.87; and HR 3.19, 95% CI 2.31–4.41; respectively). Influenza and pneumococcal immunizations were higher among SLE patients (HR 1.31, 95% CI 1.12–1.54; and HR 2.06, 95% CI 1.38–3.09; respectively), while zoster vaccination was similar (HR 1.17, 95% CI 0.81–1.69).

**Conclusions:**

The trends of utilization of preventive services by SLE patients vary according to screening or vaccine compared with the general population. Considering these differences, we demonstrate an opportunity for improvement, particularly in cervical cancer, hyperlipidemia, and osteoporosis screenings and vaccinations.

**Supplementary Information:**

The online version contains supplementary material available at 10.1186/s13075-022-02878-8.

## Background

Systemic lupus erythematosus (SLE) is a systemic autoimmune disease that affects multiple organs and systems, predominantly in women of reproductive age [1]. There is a wide range of alterations in the immune system that leads to systemic inflammation and heterogeneous organ damage in patients with SLE [2]. Disease activity is perhaps one of the main drivers of damage progression in patients with SLE [3]. Treatments, such as glucocorticoids (GC) and certain immunosuppressors (i.e., cyclophosphamide), increase the risks of damage accrual and developing cardiovascular disease (CVD), osteoporosis, infections, and cancer [4, 5]. These conditions contribute to the increased mortality observed in SLE compared to the general population [6].

The goals of treatment in patients with SLE are not only the control of signs and symptoms of the disease, but also the prevention of damage and the minimization of drugs’ side effects in order to improve long-term outcomes and patient quality of life [7]. Due to the high burden of adverse outcomes and higher demand of healthcare services compared to the general population [8, 9], it is particularly important to mitigate risks of developing conditions that are potentially preventable in order to improve the healthcare delivery and outcomes for patients with SLE [10]. Preventive services that are particularly important in this population include certain screenings for cancer, CVD, diabetes mellitus (DM), and osteoporosis as well as immunizations. It is well known that patients with SLE have an increased risk of cervical cancer [11], CVD (including hypertension and hyperlipidemia) [12], DM [12], and osteoporosis [13], as well vaccine-preventable infections [14]. While the risk of breast cancer is similar to women in the general population, the fact that most of the patients with SLE are women, and that there are established screening methods for it, make it of relevance for the SLE patient population [15, 16]. Prior studies have shown that SLE patients are assessed less frequently for CVD risk factors than non-SLE subjects [16] or patients with DM [17], despite having similar risks. Despite the described increased risks, the number of patients with SLE who get the recommended screening tests [15, 16] and vaccinations [16, 18] is suboptimal.

Screening practices are continuously evolving, and some of the previous studies may not reflect current practice; other studies were based on claims data, which could lead to misclassification, or based at academic centers where the specialized setting could differ from the real-world community setting. With these considerations, we aimed to perform a contemporary assessment of the trends of preventive services utilization in patients with SLE from the Lupus Midwest Network (LUMEN), a population-based cohort in the United States (US), compared with non-SLE subjects.

## Methods

LUMEN is a population-based registry from a 27-county region in southeast Minnesota and southwest Wisconsin, nested within the Rochester Epidemiology Project (REP) record-linkage system. The REP allows ready access to the medical records from all healthcare providers for the local population, including institutions such as Mayo Clinic, Olmsted Medical Center, their affiliated hospitals, and local nursing homes, among others. This system ensures a comprehensive ascertainment of preventive services provided among the residents of this region [19]. The demographics, distribution of morbidity, and death rates in the REP region are like those in the Upper Midwest of the US [19]. The characteristics and strengths of the REP, as well as its generalizability, have been previously described [20, 21]. This study was approved by the institutional review boards of Mayo Clinic (20-006485) and Olmsted Medical Center (036-OMC-20).

### Study population

We used the previously detailed strategies to identify, review, and abstract data from potential SLE cases [22–24]. Patients meeting the 2019 European League Against Rheumatism (EULAR)/American College of Rheumatology (ACR) classification criteria for SLE [25] were considered incident cases. Those who migrated to the 27-county region after diagnosis (and therefore were under treatment) were included if they had at least 7 EULAR/ACR points and a physician diagnosis. We included all patients meeting the requirements described above and living in the 27-county region on January 1, 2015 (index date). Patients with SLE were matched (1:1) on sex, age, race, and county to non-SLE comparators. All patients with SLE and non-SLE comparators had at least 1 year of medical history and were followed until February 29, 2020, emigration out of the geographical area, or death. February 29, 2020, was selected as the end date due to the beginning of restrictions on preventive services and non-emergency medical care secondary to the SARS-CoV-2 pandemic. Those without documented follow-up after January 1, 2015, were excluded from analyses.

### Data collection

Through medical record review, we manually abstracted demographics, disease duration, and clinical and serological SLE features. Smoking status, disease-modifying anti-rheumatic drugs (DMARDs), hyperlipidemia medications, antiosteoporotic drugs, and GC use were electronically retrieved for SLE and non-SLE comparators (see the complete list of medications in Additional file 1, supplemental table 1) for 5 years prior to index date. The patients on GC therapy were then stratified by ≥90 days and <90 days of use. Area Deprivation Index (ADI) scores at the census block group level were obtained using patient addresses [26]. These variables were evaluated at index date. We used a 5-year lookback period prior to January 1, 2015, using International Classification of Diseases Ninth Revision (ICD-9) and International Classification of Diseases Tenth Revision (ICD-10) diagnosis codes to identify the preexistence of breast, cervical, or other gynecological cancer (endometrial, uterine, placental), hypertension, hyperlipidemia, DM, and osteoporosis (see the complete list of diagnosis codes used in Additional file 1, supplemental table 2). We also calculated the Charlson Comorbidity Index [27] excluding the rheumatologic category.

### Preventive services evaluation

We evaluated breast and cervical cancer, CVD, DM, and osteoporosis screening based on the US Preventive Services Task Force (USPSTF) recommendations in effect during the 2015–2020 period [28–39]. Seasonal influenza, pneumococcal, and herpes zoster vaccination were evaluated based in accordance with the current recommendations made by the Advisory Committee on Immunization Practices from the Centers for Disease Control and Prevention in the US during the same period [40–44].

For breast cancer screening evaluation, we included all women between 50 and 74 years old (excluding those women with history of breast cancer) and assessed if they were screened with a mammogram [28, 29]. For cervical cancer screening, we included all women between 21 and 64 years old (excluding those with previous diagnosis of cervical cancer or a positive human papillomavirus [HPV] test), and assessed screening by cervical cytology (Pap smears) or polymerase chain reaction detection of HPV (high and low risk serotypes) on a cervical sample [30, 31]. As part of CVD screening, we evaluated hypertension screening in all subjects (excluding those with history of hypertension) by presence of a measurement of blood pressure [32, 33]. We also evaluated screening for hyperlipidemia with the measurement of blood lipids [34–36] in all patients (excluding those with treatments for hyperlipidemia). DM screening was evaluated in all subjects (excluding those with history of DM), by assessing the measurement of blood glucose [37]. Osteoporosis screening by dual X-ray absorptiometry (DXA) [38, 39] was evaluated in all subjects (excluding those with history of antiosteoporotic therapy), followed by stratification according to age (≥65 versus <65 years old) and to the duration of GC use (≥90 days and <90 days) due to the higher risk of developing osteoporosis at higher age and with long-term use of GC. The aforementioned measurements and tests were electronically retrieved using Current Procedural Terminology (CPT) and laboratory codes (see the complete list of codes in the Additional file 1, supplemental table 3).

Seasonal influenza vaccination was evaluated for all subjects by 12-month seasons [40], July 1 to June 30, beginning in 2015, with exception of the last season which ended on February 29, 2020 (7-month season). The evaluation of pneumococcal vaccination was performed for all subjects, assessing the 13-valent pneumococcal conjugate vaccine (PCV13) [42] and the 23-valent pneumococcal polysaccharide vaccine (PPSV23) [41]; as the age of vaccination and number of doses recommended could be different for these two vaccines, we documented the first dose of either. Herpes zoster vaccination was evaluated for all subjects ≥50 years of age by assessing the first dose of the recombinant zoster vaccine (RZV) on or after January 1, 2018; we also assessed the zoster vaccine live (ZVL) uptake before January 1, 2018 [43, 44]. The vaccination data was electronically retrieved and manually cross-checked with complementary information from the immunization information systems of Minnesota and Wisconsin.

### Statistical analysis

Descriptive statistics were used to summarize the data. Chi-square and Wilcoxon rank sum tests were performed to compare the baseline characteristics between patients with and without SLE. The cumulative incidence of screening tests and vaccination was estimated for patients with and without SLE using Kaplan-Meier methods. Cox proportional hazards models with adjustment for age, sex, and race were used to compare screening tests and vaccination rates between the two groups. Breast and cervical cancer models were adjusted for age and race as only females were included in these analyses. A *p*-value of <0.05 was considered statistically significant for all analyses. Analyses were performed using SAS version 9.4 (SAS Institute, Cary, NC, USA) and R 4.0.3 (R Foundation for Statistical Computing, Vienna, Austria).

## Results

### Baseline characteristics

There were 465 patients with prevalent SLE in the 27-county region on January 1, 2015. After matching 465 non-SLE comparators, exclusions were those age <19 years (3 SLE and 2 non-SLE), those with <1 year of prior history (11 SLE and 11 non-SLE) and those with no follow-up after January 1, 2015 (11 SLE and 22 non-SLE). The resulting study population included 440 patients with SLE and 430 non-SLE comparators. The age, sex, and racial/ethnic distribution was similar among both groups (Table 1). We did not find any difference in smoking status, body mass index, or ADI between the groups. The Charlson Comorbidity Index at baseline was higher among patients with SLE (2.3 [SD 2.6] versus 0.9 [SD 1.6], respectively, *p*<0.001), and a history of hypertension was more frequent in patients with SLE (50.2%) than comparators (35.1%, *p*<0.001). We did not find differences in the history of DM, hyperlipidemia, or breast, cervical, or any other gynecological cancer between patients with and without SLE. A history of osteoporosis was more frequent among patients with SLE than comparators (16.4% versus 4.7%, respectively, *p*<0.001), as was use of antiosteoporotic treatments. The use of hyperlipidemia therapy was similar in both groups (Table 1).

**Table 1:** Demographic and baseline characteristics of patients with systemic lupus erythematosus (SLE) and matched non-SLE comparators from the Lupus Midwest Network registry on January 1, 2015

	SLE ***N***=440	Non-SLE ***N***=430	***p***-value^a^
**Age**, years, mean (SD)	54.0 (15.6)	54.1 (15.7)	0.97
**Women**, *n* (%)	359 (81.6)	355 (82.6)	0.71
**Race/ethnicity**, *n* (%)			0.99
Non-Hispanic White	387 (88.0)	377 (87.7)	
Hispanic	19 (4.3)	17 (4.0)	
Black	13 (3.0)	15 (3.5)	
Asian	15 (3.4)	14 (3.3)	
American Indian	2 (0.5)	2 (0.5)	
Other/mixed	4 (0.9)	5 (1.2)	
**Smoking**, *n* (%)			0.15
Current^b^	83 (21.0)	90 (26.4)	
Former^b^	158 (39.9)	117 (34.3)	
Never^b^	155 (39.1)	134 (39.3)	
Missing	44	89	
**BMI**, kg/m^2^, mean (SD)	29.3 (7.5)	29.9 (7.1)	0.094
BMI ≥30kg/m^2b^, *n* (%)	161 (38.1)	155 (40.1)	0.56
BMI ≥40kg/m^2b^, *n* (%)	40 (9.5)	36 (9.3)	0.94
Missing	17	43	
**ADI**, mean (SD)	94.1 (12.6)	93.8 (12.2)	0.76
**Charlson Comorbidity Index** ^c^, mean (SD)	2.3 (2.6)	0.9 (1.6)	<0.001
**Comorbidities**, *n* (%)			
Breast cancer^d^	15 (4.2)	12 (3.4)	0.58
Cervical cancer^d^	8 (2.2)	2 (0.6)	0.058
Other gynecological cancer^d^	2 (0.6)	1 (0.3)	0.57
Hypertension	221 (50.2)	151 (35.1)	<0.001
Hyperlipidemia	173 (39.3)	164 (38.1)	0.72
Diabetes mellitus	42 (9.5)	40 (9.3)	0.90
Osteoporosis	72 (16.4)	20 (4.7)	<0.001
**Treatments**, *n* (%)			
Glucocorticoids	328 (74.5)	110 (25.6)	<0.001
≥90 days	283 (64.3)	29 (6.7)	<0.001
<90 days	45 (10.2)	81 (18.8)	<0.001
Antiosteoporotic	69 (15.7)	19 (4.4)	<0.001
Hyperlipidemia therapy	122 (27.7)	121 (28.1)	0.89

*ADI* Area Deprivation Index, *BMI* body mass index, *SD* standard deviation

^a^Wilcoxon Rank sum or chi-square test

^b^The denominator excludes missing

^c^Excluding rheumatologic category

^d^Women only (SLE=359, non-SLE=355)

Patients with SLE had a median disease duration of 10.7 years (interquartile range [IQR] 4.6–20.3). The main clinical manifestations were arthritis (65.0%) and leukopenia (42.5%); 16.1% of patients had class III/IV lupus nephritis, 74.3% were positive to anti-dsDNA and 21.1% to anti-Smith antibodies (see Additional file 1, supplemental table 4). During the 5 years prior to index, 84.8% of patients with SLE had used DMARDs; 64.3% had used GC for at least 90 days, versus 6.7% of the non-SLE comparators.

### Preventive services

#### Breast cancer screening

**Fig. 1:**
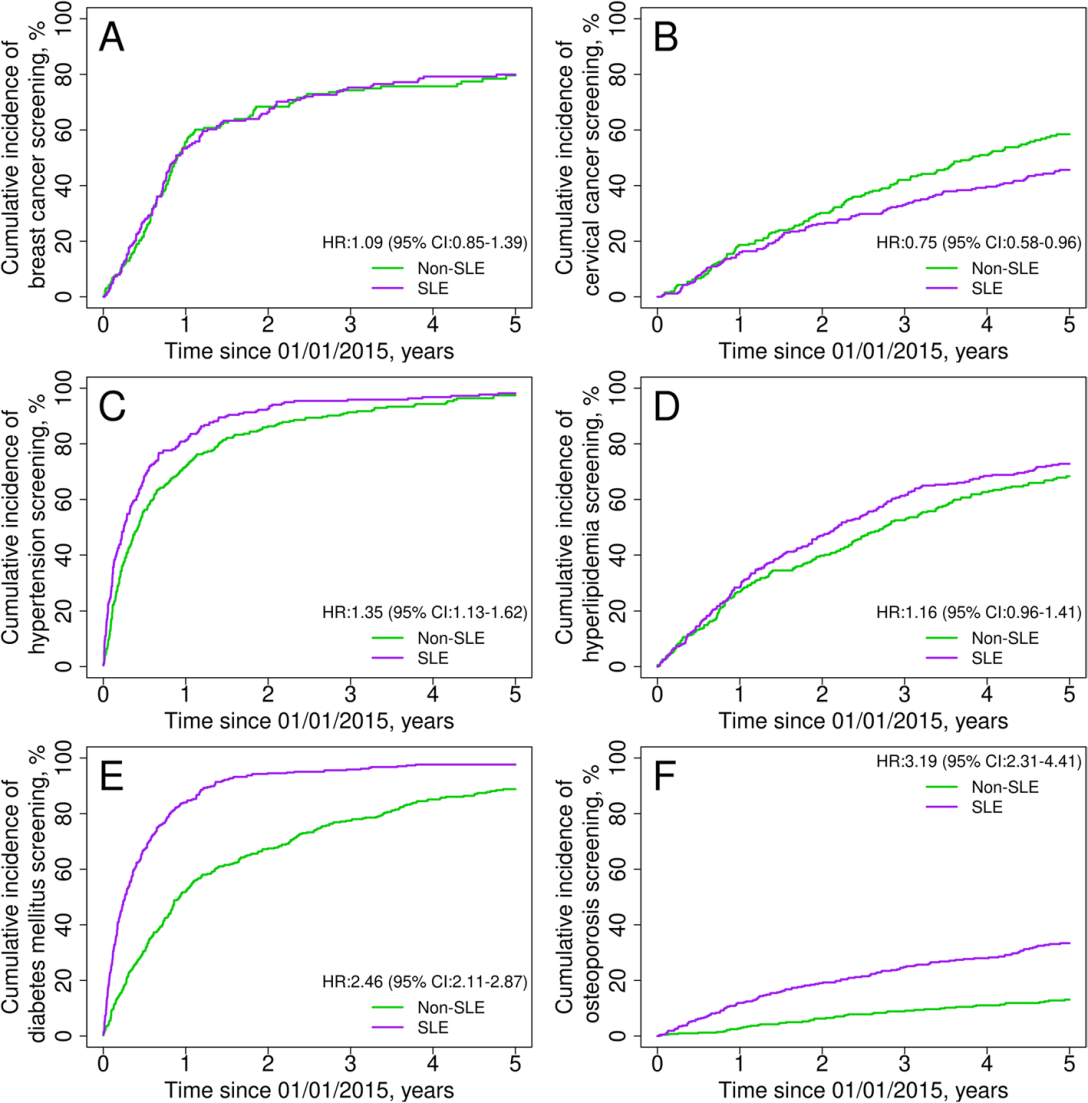
Trends and probability of receiving preventive services among systemic lupus erythematosus patients (purple line) and their comparators (green line) in the Lupus Midwest Network registry. Cumulative incidence of **A** breast cancer screening by mammograms, **B** cervical cancer screening with Pap smear or HPV test, **C** hypertension screening by office blood pressure assessment, **D** hyperlipidemia screening by blood lipids testing, **E** diabetes mellitus screening by blood glucose testing, and **F** osteoporosis screening by DXA. Hazard ratios were adjusted for age, sex, and race; those for breast and cervical cancer were adjusted for age and race

We included 164 women with SLE and 163 non-SLE comparators age 50–74 years as candidates for breast cancer screening after excluding those with a history of breast cancer. By the end of the first year of follow-up, more than half of the women with SLE (53.4%) had undergone a mammogram, with a similar proportion (55.7%) observed among non-SLE comparators (Table 2). After 5 years of follow-up, the cumulative screening remained similar in both groups (HR 1.09, 95% CI 0.85–1.39; Fig. 1A).

**Table 2:** Trends of provided preventive services in patients with and without systemic lupus erythematosus (SLE) in the Lupus Midwest Network cohort between 2015 and 2020

	Cumulative incidence, % (95% CI)						
Preventive services^a^	1 year		3 years		5 years		
	SLE	Non-SLE	SLE	Non-SLE	SLE	Non-SLE	HR^b^ (95% CI)
**Breast cancer screening**	53.4 (46.2–61.6)	55.7 (48.5–64.0)	75.2 (68.9–82.2)	74.3 (67.8–81.4)	79.9 (73.9–86.4)	79.6 (73.3–86.6)	1.09 (0.85–1.39)
**Cervical cancer screening**	16.0 (12.1–21.2)	18.7 (14.4–24.2)	33.0 (27.7–39.4)	42.1 (36.3–48.8)	45.7 (39.8–52.3)	58.5 (52.4–65.3)	0.75 (0.58–0.96)
**Hypertension screening**	81.3 (76.3–86.6)	71.8 (66.7–77.3)	95.9 (93.3–98.6)	91.3 (88.0–94.8)	98.2 (96.4–99.9)	97.4 (95.3–99.6)	1.35 (1.13–1.62)
**Hyperlipidemia screening**	28.4 (23.9–33.9)	26.9 (22.3–32.3)	61.3 (56.1–67.0)	52.5 (47.1–58.5)	72.8 (67.9–78.0)	68.3 (63.0–74.1)	1.16 (0.96–1.41)
**Diabetes mellitus screening**	84.0 (80.4–87.7)	52.0 (47.2–57.2)	95.9 (93.9–97.9)	77.4 (73.3–81.8)	97.6 (96.1–99.2)	88.8 (85.5–92.2)	2.46 (2.11–2.87)
**Osteoporosis screening**	11.9 (9.0–15.7)	2.7 (1.5–4.9)	24.8 (20.8–29.7)	8.9 (6.5–12.2)	33.4 (28.8–38.7)	13.1 (10.1–17.0)	3.19 (2.31–4.41)
Age ≥65 years old	16.5 (10.2–26.7)	5.2 (2.2–12.1)	32.0 (23.5–43.7)	22.0 (15.1–32.2)	39.4 (30.2–51.4)	28.7 (20.9–39.5)	1.65 (1.00–2.73)
Age <65 years old	10.6 (7.5–14.8)	1.9 (0.9–4.3)	22.7 (18.3–28.2)	4.7 (2.8–7.8)	31.6 (26.5–37.6)	8.1 (5.4–12.0)	5.27 (3.35–8.29)
Glucocorticoid use^c^							
≥90 days	12.7 (9.1–17.9)	3.8 (0.6–26.3)	25.2 (20.2–31.6)	11.9 (4.1–34.3)	34.4 (28.7–41.3)	16.1 (6.5–39.5)	2.55 (0.93–6.98)
<90 days	10.6 (6.6 –17.2)	2.6 (1.4–4.9)	24.2 (18.1–32.4)	8.7 (6.2–12.1)	31.8 (24.9–40.6)	12.9 (9.8–16.9)	3.23 (2.14–4.87)

*CI* confidence interval, *HR* hazard ratio

^a^Breast cancer screening was evaluated with mammograms; the recommended interval was every 2 years [28, 29]. Cervical cancer screening was evaluated with Pap smears and/or HPV tests; the recommended interval was every 3 years with Pap smear or every 5 years with HPV test [30, 31]. Hypertension screening was evaluated with office measurement of blood pressure; the recommended interval ranges from yearly in those aged 40 years or older or at increased risk, to every 3 to 5 years in those younger than 40 years with an initial normal blood pressure (<130/85 mmHg) and without risk factors [32, 33]. Hyperlipidemia screening was evaluated with the measurement of blood lipids; there was not an established recommended interval [34–36]. Diabetes mellitus screening was evaluated with the measurement of blood glucose; the recommended interval was every 3 years [37]. Osteoporosis screening was evaluated with dual X-ray absorptiometry; the recommended interval was uncertain [38, 39]

^b^Cox proportional hazards models at 5 years with adjustment for age, sex, and race; and age and race for women only screenings. The number of patients at risk on each timepoint is shown in supplemental table 5

^c^At index date (January 1, 2015)

#### Cervical cancer screening

After excluding those with a history of cervical cancer, we included 257 women with SLE and 256 comparators age 21–64 years as candidates for cervical cancer screening. During the first 2 years of follow-up, SLE and non-SLE women had similar trends in Pap smears and HPV testing (Fig. 1B). However, by the third and fifth years, these tests were a quarter lower in patients with SLE compared with those without SLE (HR 0.75, 95% CI 0.58–0.96; Table 2).

#### Hypertension screening

We included 219 patients with SLE and 279 comparators for the hypertension screening evaluation, after excluding those with a history of hypertension. Blood pressure assessment was more frequently done among patients with SLE than non-SLE comparators (Table 2); patients with SLE were 35% more likely to be screened for hypertension than the comparators without SLE during the 5 years evaluated (HR 1.35, 95% CI 1.13–1.62; Fig. 1C).

#### Hyperlipidemia screening

After excluding those with hyperlipidemia-related prescriptions within the 5 years prior to index, 318 patients with SLE and 309 comparators were included for the hyperlipidemia screening evaluation. Blood lipids testing among patients with SLE and comparators was no different during the 5 years of observation (HR 1.16, 95% CI 0.96–1.41; Fig. 1D).

#### Diabetes mellitus screening

We included 398 patients with SLE and 390 non-SLE comparators. After excluding patients with a history of DM, patients with SLE were more than twice as likely to be screened for DM than non-SLE comparators (HR 2.46, 95% CI 2.11–2.87; Table 2). While more than 80% of patients with SLE were screened within 1 year (Fig. 1E), it took more than 3 years to reach this same proportion of screened patients in the general population.

#### Osteoporosis screening

We included 371 patients with SLE and 411 comparators after excluding those receiving antiosteoporotic treatments in the 5 years prior to index. Osteoporosis screening with DXA was more frequent among patients with SLE than the comparator population throughout the 5 years of observation (Table 2), and they were more than three times as likely to be screened compared with the non-SLE subjects (HR 3.19, 95% CI 2.31–4.41) (Fig. 1F). Men were less likely screened than women (HR 0.22, 95% CI 0.12–0.41). Patients with SLE <65 years of age were more than five times as likely to be screened as their counterparts (HR 5.27, 95% CI 3.35–8.29), patients with SLE ≥65 years of age were also more likely to be screened (HR 1.65, 95% CI 1.00–2.73). When we compared patients with SLE who used GC for ≥90 days versus <90 days during the 5 years preceding index, we did not find any difference in osteoporosis screening trends.

#### Immunizations

##### Seasonal influenza vaccine

**Fig. 2:**
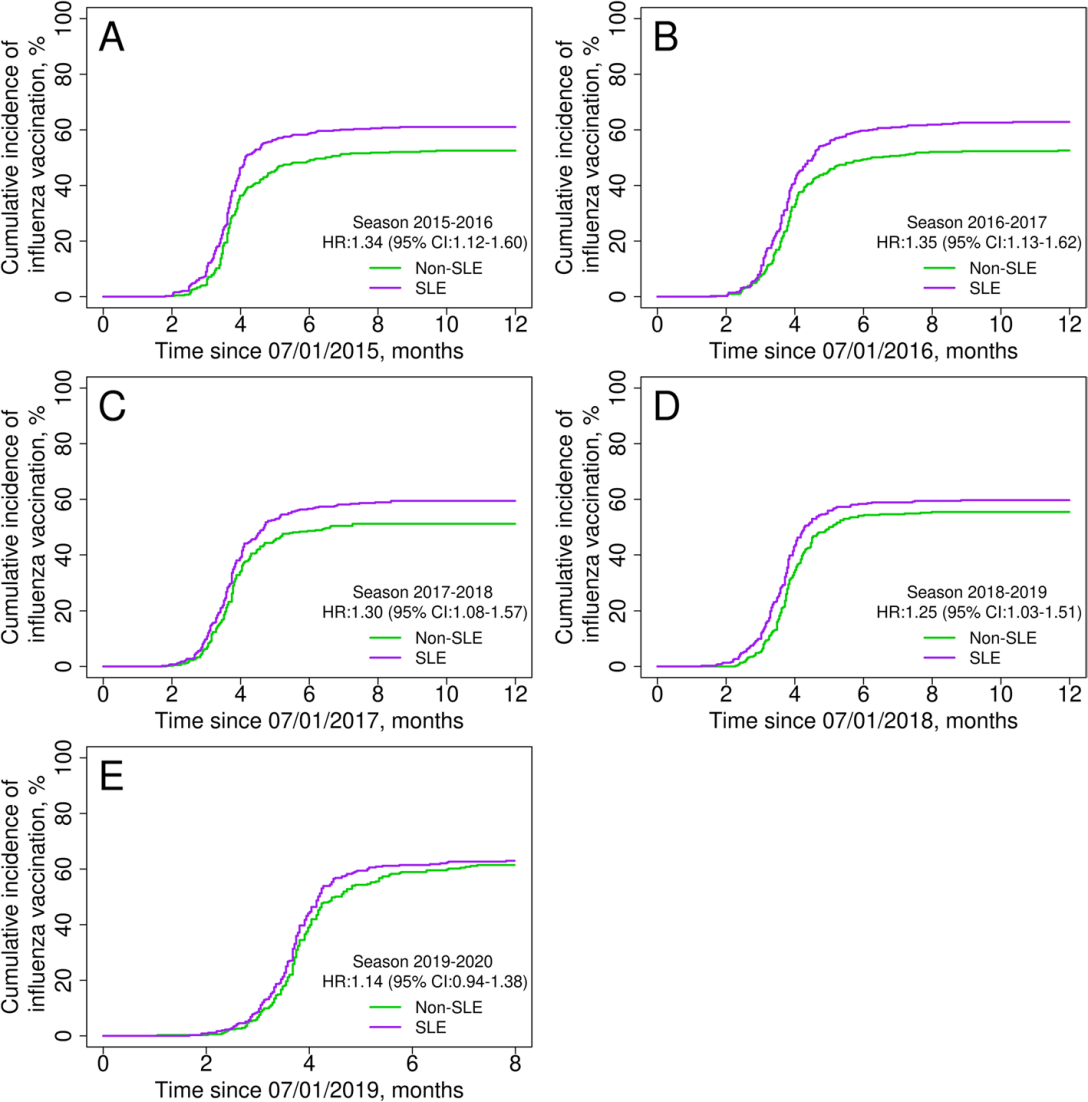
Seasonal influenza vaccine uptake among systemic lupus erythematosus patients (purple line) and comparators (green line) during five consecutive vaccination seasons in the Lupus Midwest Network registry. Hazard ratios were adjusted for age, sex, and race

Patients with SLE were 30% more likely than the general population to get at least one seasonal influenza vaccination during a five-season period of observation (HR 1.31, 95% CI 1.12–1.54; Table 3). During the five individual seasons evaluated in our cohort, the yearly influenza vaccination rate ranged between 59.4% (in 2017–2018) and 63.0% (in 2019–2020) among patients with SLE, and between 51.2% (in 2017–2018) and 61.5% (in 2019–2020) among non-SLE comparators (Table 4). Most patients (SLE and non-SLE) received their influenza vaccine within the first 3 months of availability in each season (Fig. 2).

**Table 3:** Trends of vaccine uptake in patients with and without systemic lupus erythematosus (SLE) in the Lupus Midwest Network cohort between 2015 and 2020

	Cumulative incidence, % (95% CI)								
Immunization^a^	1 year		2 years		3 years		5 years		
	SLE	Non-SLE	SLE	Non-SLE	SLE	Non-SLE	SLE	Non-SLE	HR^b^ (95% CI)
Influenza	60.6 (56.2–65.3)	51.6 (47.1–56.6)	69.4 (65.2–73.8)	60.1 (55.6–65.0)	73.0 (68.9–77.3)	63.5 (59.0–68.3)	75.3 (71.3–79.5)	69.8 (65.4–74.6)	1.31 (1.12–1.54)
Pneumococcal disease	6.9 (3.8–12.5)	4.1 (2.3–7.4)	12.5 (8.1–19.2)	9.2 (6.3–13.5)	25.5 (19.2–33.8)	12.8 (9.3–17.6)	33.9 (26.8–42.8)	18.2 (13.9–23.7)	2.06 (1.38–3.09)
Herpes zoster^c^	11.4 (7.9–16.5)	7.5 (4.8–11.9)	25.8 (20.6–32.4)	22.3 (17.3–28.6)	–	–	–	–	1.17 (0.81–1.69)

*CI* confidence interval, HR hazard ratio

^a^Seasonal influenza immunization was recommended every year [40]. Pneumococcal immunization was evaluated with the 13-valent pneumococcal conjugate vaccine (PCV13) and the 23-valent pneumococcal polysaccharide vaccine (PPSV23); the recommended revaccination for the PPSV 23 was 5 years after the first dose, up to 2 shots in lifetime, and there was not a revaccination recommendation for the PCV13 [41, 42]. Zoster vaccination was evaluated with a single dose of the recombinant zoster vaccine; a second dose should be given 2–6 months later, up to 2 shots in lifetime [43, 44]

^b^Cox proportional hazards models with adjustment for age, sex, and race at 5 or 2 years, as correspond. The number of patients at risk on each timepoint is shown in supplemental table 6

^c^After recombinant zoster vaccine became available (January 1, 2018)

**Table 4:** Cumulative incidence of influenza vaccine uptake by season between July 2015 and February 2020 among patients with systemic lupus erythematosus (SLE) and matched comparators in the Lupus Midwest Network cohort

	Cumulative incidence, % (95% CI)		
Influenza season	SLE	Non-SLE	HR^a^ (95% CI)
2015–2016	61.0 (56.6–65.8)	52.5 (48.0–57.5)	1.34 (1.12–1.60)
2016–2017	62.8 (58.4–67.7)	52.6 (48.0–57.6)	1.35 (1.13–1.62)
2017–2018	59.4 (54.8–64.5)	51.2 (46.5–56.4)	1.30 (1.08–1.57)
2018–2019	59.7 (54.9–64.9)	55.4 (50.6–60.7)	1.25 (1.03–1.51)
2019–2020^b^	63.0 (58.1–68.3)	61.5 (56.4–66.9)	1.14 (0.94–1.38)

*CI* confidence interval, *HR* hazard ratio

^a^Cox proportional hazards models with adjustment for age, sex, and race at the end of each season

^b^For this study, this season ended on February 29, 2020, due to the beginning of restrictions secondary to SARS-CoV-2 pandemic

##### Pneumococcal vaccine

**Fig. 3:**
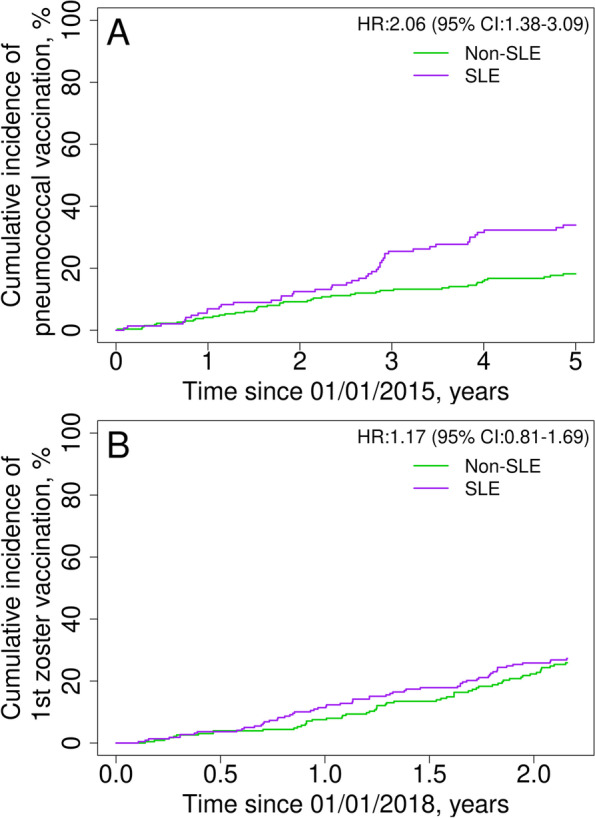
Cumulative incidence of **A** pneumococcal and **B** zoster vaccination uptake among patients with systemic lupus erythematosus (purple line) and comparators (green line) in the LUMEN registry. Hazard ratios were adjusted for age, sex, and race

Excluding those patients who were vaccinated before January 1, 2015, the pneumococcal vaccination rate among patients with SLE was twice that of the general population during the 5-year period of follow-up (HR 2.06, 95% CI 1.38–3.09; Table 3 and Fig. 3A). When including those vaccinated prior to January 1, 2015, 78.1% of patients with SLE and 48.4% of non-SLE comparators were vaccinated against *Pneumococcus* at least once by January 1, 2020.

##### Herpes zoster vaccine

Before 2018, among the patients with SLE and the non-SLE comparators 50 years or older, the ZVL uptake was lower in patients with SLE (18.0%) compared with the non-SLE population (25.1%, *p*=0.010). Once the RZV was available, the zoster vaccination uptake was similar in patients with SLE and comparators (HR 1.17, 95% CI 0.81–1.69; Table 3 and Fig. 3B). By February 29, 2020, 27.3% of patients with SLE 50 years old or older were vaccinated with RZV versus 25.9% of non-SLE comparators.

## Discussion

In this study, we described our findings on the utilization of preventive services among patients with SLE and their matched non-SLE comparators. The implementation of preventive services is impactful in the care of patients with SLE due to the higher risk of developing adverse outcomes after the beginning of the disease [4–6]. While breast cancer screening among women with SLE was similar to the general population, cervical cancer screening was lower in women with SLE than in the comparators, despite the increased risk of this cancer in women with SLE. In contrast, hypertension screening through the office measurement of blood pressure was higher among patients with SLE, although hyperlipidemia screening through blood lipids testing was similar to matched comparators. Screening for DM and for osteoporosis was two and three times more likely, respectively, to be performed on patients with SLE than for comparators. The trend of influenza immunization was slightly higher among patients with SLE throughout the five-season period of evaluation, as well as when broken down by individual season. Regarding pneumococcal vaccination, patients with SLE were twice as likely to be vaccinated at least once during the last 5 years. ZVL was not recommended in persons under moderate to high doses of immunosuppressive therapy [44]. With the availability of RZV, zoster vaccination in patients with SLE was similar to vaccination in non-SLE counterparts—an improvement from being significantly below the general population with the previous ZVL. After this comprehensive evaluation based on the US regulatory agencies recommendations, we found that patients with SLE showed a different utilization of preventive services compared with the general population.

We found that more than half of the women with SLE 50 to 74 years old with no history of breast cancer had a mammogram during the first year of follow-up, and after 5 years, four out of five women with SLE had been screened for breast cancer at least once. This trend was also observed in non-SLE comparators. In a previous survey between 2004 and 2005 from the Montreal General Hospital lupus cohort, they reported that half of the women with SLE aged 50–69 years had undergone mammography in the previous 12 months, which was lower than the rate in their general population [45]. Another study between 2005 and 2006, derived from the Lupus Outcomes Study (LOS) based in the University of California, San Francisco, reported that among women with SLE ≥40 years old, 70% had received a mammogram over the previous year of the survey, similar to their general population [15]. A cross-sectional study from the Georgians Organized Against Lupus (GOAL) cohort reported that 84.3% of patients with SLE had received a mammogram within the previous 2 years of survey, higher than their comparative community sample [16]. Some methodological and population-based differences could explain our different findings. We used the REP infrastructure, allowing us to ascertain the documented fulfillment of the testing instead of survey data with inherent reporting limitations. These other studies had different targeted age populations since the recommendations were different; we selected the age range based on the USPSTF recommendations in effect during the period evaluated. Additionally, the racial/ethnical distribution of the LUMEN, LOS, and GOAL cohorts differed.

While breast cancer screening is recommended every 1–2 years [28, 29], cervical cancer screening, through Pap smear and/or HPV test, is recommended every 3–5 years [30, 31]. Our study found that one out of six women with SLE (16.0%), ages 21–64 years with no history of cervical cancer, was screened within 1 year, and by the end of the third year, one out of three women with SLE (33.0%) had undergone a cervical cancer screening. These findings were similar in the matched general population during the first 2 years, but over the course of the 5 years of follow-up, the cumulative incidence of screening was lower among SLE patients. In the study by Bernatsky et al., they found that 43.8% of SLE women had reported a Pap smear in the preceding 12 months, lower than their comparators [45]. Patients with SLE may miss some of their preventive screenings due to the complexity of their care. The study from the LOS cohort, found that 70% of the women with SLE aged 18–65 years old had self-reported cervical cancer screening during the previous year of the study, similar to what was reported by their general population group [15]. The GOAL cohort study found that 83.2% of their women with SLE aged 18–65 years old had received a Pap smear in the past 2 years before the survey, also similar to their comparators [16]. A recent US claims-based study, including women with incident SLE aged 21–64 years, described that 73.4% of women with SLE had undergone cervical cancer screening within 1 year prior to and 2 years after a medical claim related to SLE, exceeding the rates of the matched comparators [46]. As was previously mentioned, some of these differences could be explained by the different methods, population characteristics, and recommendations which were current during these studies. Due to the higher risk of cervical neoplasia described in women with SLE [11], strategies to improve screening rates should be developed.

CVD is one of the leading causes of death among patients with SLE [6]. Surprisingly, almost 20% of patients with SLE without a history of hypertension did not have any blood pressure measurements taken, compared to nearly 30% of matched comparators, during the first year of follow-up. This proportion decreased to <5% by the third year and <2% after 5 years among patients with SLE; comparators were at about 14% and 9% at 3 and 5 years, respectively. A previous Canadian study, based on medical records review, showed that 26% of SLE patients did not have a documented blood pressure measurement at the initial visit to their clinic [47]. A recent claims-based study, derived from the ACR’s Rheumatology Informatics System for Effectiveness (RISE) registry, described that 94.4% of patients with SLE had a blood pressure assessment during a calendar year period [48]. Neither of the aforementioned studies excluded patients with hypertension, as our study did, and also theirs were based on rheumatology practices while our study looked at the healthcare system; thus, it was difficult to compare our results with theirs.

In our assessment of blood lipids, we found the rate of testing was similar among patients with SLE and non-SLE comparators with almost 30% during the first year and roughly 70% after 5 years in both groups. The previously mentioned Canadian study found that 31% of patients with SLE had at least one lipid test performed at their initial visit [47], a similar rate to ours at 1 year. The GOAL cohort study also evaluated lipid monitoring for all patients with SLE, finding that 65% had the lipid levels measured within the previous year, which was significantly lower than that of their comparators with CVD risk factors [16] and similar to our rate after 3 years. An important difference to highlight between these studies and ours was our exclusion of patients who were using hyperlipidemia-related therapy; studies without this exclusion would have higher blood lipid monitoring rates due to assessment of treatment efficacy, while we focused on primary screening.

Among patients without a history of DM, we found that patients with SLE were almost two and a half times more likely to be screened for DM than matched comparators, and most were screened during the first 2 years of follow-up. There is a scarcity of reports exploring this topic. Al-Herz et al. in Canada described that only 51% of patients with SLE had a serum blood glucose measurement documented in their medical record [47]. To the best of our knowledge, we are the first to explore primary screening for DM among patients with SLE compared to the general population. The higher rates of screening among patients with SLE could be explained by more frequent healthcare utilization [8, 9, 49] as well as the wide availability of blood glucose testing in any setting.

Patients with SLE were three times as likely to be screened for osteoporosis than non-SLE comparators, with similar trends observed when separated by duration of GC therapy; patients with SLE <65 years of age, however, were more than five times as likely to be screened as their non-SLE counterparts. By the end of the fifth year of follow-up, one third of patients with SLE had been screened for osteoporosis. A cross-sectional study reported that 33.5% of patients with SLE had a DXA test performed during the previous 2 years, and interestingly they reported more patients under antiosteoporotic therapy than were DXA-tested [50]. We cannot compare our findings with this report because we excluded all patients using antiosteoporotic therapy. It should be noted that we decided to include all patients at least 19 years of age instead of only those 65 years or older as is recommended for the general population [38, 39] because it is known that, besides inflammation, there are multiple additional risk factors for patients with SLE which lead to an increased risk of osteoporosis [13]. Nevertheless, this inclusion could overestimate the testing rates among patients with SLE compared to the general population, where testing is not recommended before the age of 65 years. However, for those 65 years or older, the estimates were consistently higher during the first 3 years among patients with SLE compared to non-SLE comparators. Efforts to increase the awareness of osteoporosis screening among patients with SLE, and particularly for those with longer use of GC, are needed.

Around 60% of patients with SLE were vaccinated against influenza during each of the five seasons evaluated, slightly higher than the non-SLE comparators. Interestingly, after five seasons nearly 25% of patients with SLE had never been vaccinated against influenza. Pneumococcal vaccination among patients with SLE was nearly 34% after 5 years of follow-up—almost twice that of the general population. Zoster vaccination with the RZV vaccine was not statistically different between the two groups. The studies from the LOS [15] and GOAL [16] cohorts reported an influenza vaccination uptake among patients with SLE of 59 and 57.1%, and a pneumococcal vaccination uptake of 60 and 49.1%, respectively. In both studies, the uptake was higher compared with their general population. Although the recommendations during these studies were different, our findings are concordant regarding the influenza vaccine but lower regarding the pneumococcal vaccine. As was previously mentioned, there are several differences that could explain the variation in results.

Patients with SLE are at increased risk of herpes zoster infection [14]; however, there is only one study of zoster vaccination of patients with SLE which was performed when only the ZVL was available; this study reported an uptake of 7.1% among age-eligible patients with SLE, which was lower than their comparators [51]. To the best of our knowledge, this is the first study reporting zoster vaccine uptake among patients with SLE and comparators since the RZV became available.

Some strengths of our study are due to the population-based design nested in the REP, which allowed us to depict the real state of SLE care compared with the general population. This same infrastructure helped us to limit the risks of retrieval, reporting, and recall biases. Our report is up to date until just prior to the SARS-CoV-2 pandemic, which led to many changes in current healthcare practice. To avoid the risk of overestimation by including subjects with a history of any of the focused conditions, for each evaluated screening test we selected only the population of those who were candidates for that specific testing. Nevertheless, our study has some limitations: Our study population may not be generalizable to other populations with more racial/ethnic diversity. Patients with SLE have more medical encounters than those without SLE, these encounters are in its majority with subspecialists who may or may not address primary care needs. Our study was not designed to assess the impact of subspecialty visits on preventive services utilization, and this warrants further study. Screenings made in other geographical areas might not be documented.

## Conclusions

Patients with SLE presented different trends in preventive service utilization. While most of the preventive services were performed at least as frequently as in the general population, others like cervical cancer screening were lower despite the higher risk for cervical neoplasia in patients with SLE. However, we should not feel complacent by having similar rates of preventive services between patients with SLE and the general population, since most of the diseases targeted by these screening methods are more frequent and/or a leading cause of death in SLE. Our results provide a contemporary survey of the utilization of preventive services among patients with SLE and demonstrate an opportunity for improvement, particularly in cervical cancer, lipids, and osteoporosis screenings and vaccinations.

## Supplementary Information

**Additional file 1: Supplemental table 1.** List of medications electronically evaluated in patients with and without systemic lupus erythematosus grouped by type. **Supplemental table 2.** International Classification of Diseases, Ninth (ICD-9) and Tenth (ICD-10) Revision codes used to identify comorbidities. **Supplemental table 3.** Current Procedural Terminology codes used to identify the measurements and tests for screening along with the vaccination status. **Supplemental table 4.** Clinical manifestations and organ involvement of patients with systemic lupus erythematosus (SLE) from the Lupus Midwest Network cohort at or ever prior to January 1, 2015. **Supplemental table 5.** Number of patients with and without systemic lupus erythematosus (SLE) at risk at each timepoint during the assessment of preventive services in the Lupus Midwest Network cohort between 2015 and 2020. **Supplemental table 6.** Number of patients with and without systemic lupus erythematosus (SLE) at risk at each timepoint during the assessment of immunizations in the Lupus Midwest Network cohort between 2015 and 2020.

## Data Availability

The datasets used and/or analyzed during the current study are available from the corresponding author on reasonable request and ethical approval.

## Abbreviations

ACR: American College of Rheumatology
ADI: Area Deprivation Index
Anti-dsDNA: Anti-double-stranded DNA
BMI: Body mass index
CI: Confidence interval
CPT: Current Procedural Terminology
CVD: Cardiovascular disease
DM: Diabetes mellitus
DMARDs: Disease-modifying anti-rheumatic drugs
DXA: Dual X-ray absorptiometry
EULAR: European League Against Rheumatism
GC: Glucocorticoids
GOAL: Georgians Organized Against Lupus
HPV: Human papillomavirus
HR: Hazard ratio
ICD: International Classification of Diseases
IQR: Interquartile range
LOS: Lupus Outcomes Study
LUMEN: Lupus Midwest Network
PCV13: 13-valent pneumococcal conjugate vaccine
PPSV23: 23-valent pneumococcal polysaccharide vaccine
REP: Rochester Epidemiology Project
RISE: Rheumatology Informatics System for Effectiveness
RZV: Recombinant zoster vaccine
SD: Standard deviation
SLE: Systemic lupus erythematosus
US: United States
USPSTF: United States Preventive Services Task Force
ZVL: Zoster vaccine live
