# Supplementary material for: Utilization of preventive services in a systemic lupus erythematosus population-based cohort: a Lupus Midwest Network (LUMEN) study
Source: Arthritis Res Ther. 2022 Sep 1;24:211. doi: 10.1186/s13075-022-02878-8 (PMC9434086; doi:10.1186/s13075-022-02878-8)
Supplement: Supplementary file 1 — Additional file 1: Supplemental table 1. List of medications electronically evaluated in patients with and without systemic lupus erythematosus grouped by type. Supplemental table 2. International Classification of Diseases, Ninth (ICD-9) and Tenth (ICD-10) Revision codes used to identify comorbidities. Supplemental table 3. Current Procedural Terminology codes used to identify the measurements and tests for screening along with the vaccination status. Supplemental table 4. Clinical manifestations and organ involvement of patients with systemic lupus erythematosus (SLE) from the Lupus Midwest Network cohort at or ever prior to January 1, 2015. Supplemental table 5. Number of patients with and without systemic lupus erythematosus (SLE) at risk at each timepoint during the assessment of preventive services in the Lupus Midwest Network cohort between 2015 and 2020. Supplemental table 6. Number of patients with and without systemic lupus erythematosus (SLE) at risk at each timepoint during the assessment of immunizations in the Lupus Midwest Network cohort between 2015 and 2020. [file 13075_2022_2878_MOESM1_ESM.docx]

**Supplemental table 1.** List of medications electronically evaluated in patients with and without systemic lupus erythematosus grouped by type.

| **Type** | **Name** |
| --- | --- |
| Antiosteoporotic drugs | TERIPARATIDE, ALENDRONATE, ALENDRONIC ACID, IBANDRONATE, PAMIDRONIC ACID, RALOXIFENE, RISEDRONATE, ZOLEDRONIC ACID, DENOSUMAB |
| Disease modifying anti-rheumatic drugs | CYCLOPHOSPHAMIDE, METHOTREXATE, RITUXIMAB, CHLOROQUINE, HYDROXYCHLOROQUINE, CERTOLIZUMAB PEGOL, SULFASALAZINE, AZATHIOPRINE, CANAKINUMAB, CYCLOSPORINE, INFLIXIMAB, MYCOPHENOLATE MOFETIL, MYCOPHENOLIC ACID, TACROLIMUS, USTEKINUMAB, BELIMUMAB, ABATACEPT, ADALIMUMAB, ANAKINRA, ETANERCEPT, GOLIMUMAB, LEFLUNOMIDE, TOCILIZUMAB |
| Hyperlipidemia-related drugs | EZETIMIBE, EZETIMIBE / SIMVASTATIN, FENOFIBRATE, FLUVASTATIN, GEMFIBROZIL, LOVASTATIN, PRAVASTATIN, ROSUVASTATIN, SIMVASTATIN, ATORVASTATIN |
| Glucocorticoids | CORTISONE, DEXAMETHASONE, HYDROCORTISONE, METHYLPREDNISOLONE, PREDNISOLONE, PREDNISONE, TRIAMCINOLONE |

**Supplemental table 2.** International Classification of Diseases, Ninth (ICD-9) and Tenth (ICD-10) Revision codes used to identify comorbidities.

| **Condition** | **ICD-9 codes** | **ICD-10 codes** |
| --- | --- | --- |
| Breast cancer | 174.x, 175.x, V10.3, 233.0 | C50.x, Z85.3, D05.x |
| Cervical cancer | 180.x, V10.41, 233.1 | C53.x, Z85.41, D06.x |
| Other gynecological cancer | 179, 181, 182.x, V10.42, 233.2 | C54.x, C55, C58, Z485,42, D07.0 |
| Hypertension | 401.x, 402, 402.0, 402.00, 402.1, 402.10, 402.9, 402.90, 403, 403.0, 403.00, 403.1, 403.10, 403.9, 403.90, 404, 404.0, 404.00, 404.1, 404.10, 404.9, 404.90, 405.x | I10.x, I11.x, I12.x, I13.x, I15.x, I16.x |
| Hyperlipidemia | 272.0, 272.2, 272.3, 272.4 | E78.0x, E78.2, E78.3, E78.4x, E78.5 |
| Diabetes mellitus | 250.x | E10.x, E11.x, E13.x, E14.x |
| Osteoporosis | 733.0x, 733.1x | M80.x, M81.x |

**Supplemental table 3.** Current Procedural Terminology codes used to identify the measurements and tests for screening along with the vaccination status.

| **Test/Procedure/Vaccine** | **CPT codes** |
| --- | --- |
| Mammograms | 76090, 76091, 76092, 76093, 76094, 76095, 76096, 77032, 77046, 77047, 77048, 77049, 77051, 77052, 77053, 77054, 77055, 77056, 77057, 77058, 77059, 77061, 77062, 77063, 77065, 77066, 77067, G0206, G0236, G8111, G8112, G8873, S8075, 19281, 19282, 19283, 19284, 19285, 19286, 19287, 19288, 19290, 19291, 19294, 19295, 19296, 19297, 19298, 3340F, 3341F, 3342F, 3343F, 3344F, 3345F, 3350F, 5060F, 5062F, 7020F, 7025F, 76082, 76083, 76085, 76086, 76088, G0202, G0203, G0204, G0205, G0207, G9899, G9900, G0279 |
| Cervical cytology (Pap smear) | 88141, 88142, 88143, 88144, 88145, 88147, 88148, 88150, 88151, 88152, 88153, 88154, 88155, 88156, 88157, 88158, 88164, 88165, 88166, 88167, 88174, 88175, G0123, G0124, G0141, G0143, G0144, G0145, G0147, G0148, P3000, P3001, Q0091 |
| HPV test | 35926-ROCMIC, 21782-ROCLIS, 21782-ROCLIS, 21783-ROCLIS, 21783-ROCLIS, 21845-ROCLIS, 21845-ROCLIS, 35924-ROCMIC, 35925-ROCMIC, 83344-ROCMIC, 1162938215, 1162938269, 2324505573, 27176242, 5189, 5190, 5191, 13536, 13537, 13538, 22686, 22687, 22688 |
| Blood pressure (vital measure) | 15627, 30, 39, 6004, 7226597, 7227313, 7228593, 7229601, 7230604, 7230605, 7230619, 7230620, 7231312, 7231605, 7231606, 7231607, 7463552, 7466553, 7468552, 7645557, 9664, BP_DIASTOLIC, BP_POSITION, BP_SYSTOLIC |
| Blood lipids test | 8320-ROCLIS, 15221-ROCLIS, 21087-ROCLIS, 4421848, CHOL, 15222-ROCLIS, 8316-ROCLIS, 21090-ROCLIS, 4728603, TRIG, 15223-ROCLIS, 8429-ROCLIS, 82024-ROCLIS, 28054631, HDL, 2357-ROCLIS, 15224-ROCLIS, 9342-ROCLIS, 80198-ROCLIS, 21093-ROCLIS, 27174745, LDL, 1552156, 1557760, 1557762, 1726268, 1811467, 28149, 28153, 28155, 28156, 4421505, 4425422, 4425957 |
| Blood glucose test | 10086, 11478, 11907, 11908, 11923, 11936, 11937, 11975, 12097, 13342, 13636, 13727, 1510655, 1510656, 1510883, 1511112, 1526530, 1534354, 1534355, 1534393, 1537859, 1552105, 1558033, 1558034, 1558035, 1558036, 1558037, 1558038, 1558041, 1558043, 1558048, 1724335, 1810177, 1810566, 1910200034, 1910200044, 1910200045, 1910200046, 1910200047, 1910200254, 1910200274, 1910200322, 1910200342, 1910200368, 1910204894, 1910205411, 22430, 22451, 22767, 23458, 24177, 24717, 24718, 24720, 24721, 24722, 24723, 24724, 24725, 24726, 24727, 24728, 24729, 24730, 24731, 24732, 24734, 24735, 24736, 24738, 24740, 24741, 24742, 24743, 24744, 24745, 24746, 24747, 24748, 24749, 24751, 24752, 24753, 24754, 24755, 24756, 24757, 24758, 24773, 24774, 24775, 24776, 24777, 24779, 24780, 24781, 24782, 24783, 24784, 24785, 24881, 24886, 25125, 25126, 25191, 25194, 25290, 25291, 25292, 25293, 25294, 25295, 25378, 3005220, 3005227, 3005236, 4565, 4833, 4834, 4835, 4836, 4837, 4838, 4839, 4840, 4841, 4842, 4843, 4846, 4847, 7662, 879, 885, 903, 904, 905, 9372, 9680, 24719, 24733, 24739, 24750, 24915, 25253, 25254, 25255, 25256, 25257, 25278, 25279, 25280, 25281, 27100, 27787, 27788, 27906, 27907, 27908, 27909, 27910, 27911, 27912, 28541, 28542, 28543, 28544, 28545, 28547, 28548, 29522, 1810144, 1810145, 1810175, 3000888, 1910205902, 3000029289, 3000029290, 3000029522, 3000029523, 3001000488, 3001000491, 20000004512, 10100719, 1347442575, 2270613, 2508417, 2509669, 2509670, 2511689, 2512182, 300650781, 348532129, 348533019, 348543522, 348543572, 357745665, 366477700, 424932575, 445499314, 445499319, 445499324, 445499329, 445499334, 552214684, 552214689, 552214694, 552214699, 552214704, 552214709, 552214719, 552214729, 552214734, 552214739, 552214744, 0.5BS, 1.5BS, 13094-ROCLIS, 13166-ROCLIS, 15044-ROCLIS, 152-ROCLIS, 15565-ROCLIS, 15577-ROCLIS, 15583-ROCLIS, 15596-ROCLIS, 1BS, 21288-ROCLIS, 2153-ROCLIS, 2178-ROCLIS, 2372-ROCLIS, 28016-ROCLIS, 28130-ROCLIS, 28133-ROCLIS, 28171-ROCLIS, 28173-ROCLIS, 28175-ROCLIS, 28177-ROCLIS, 28179-ROCLIS, 28180-ROCLIS, 28181-ROCLIS, 28568-ROCLIS, 28932-ROCLIS, 28933-ROCLIS, 28934-ROCLIS, 28935-ROCLIS, 29031-ROCLIS, 29878-ROCLIS, 2998-ROCLIS, 2BS, 3045-ROCLIS, 3145-ROCLIS, 3244-ROCLIS, 3269-ROCLIS, 3621-ROCLIS, 3622-ROCLIS, 3623-ROCLIS, 3624-ROCLIS, 3625-ROCLIS, 3666-ROCLIS, 3667-ROCLIS, 3668-ROCLIS, 3676-ROCLIS, 3677-ROCLIS, 3745-, ROCLIS, 3746-ROCLIS, 3747-ROCLIS, 3748-ROCLIS, 3749-ROCLIS, 3750-ROCLIS, 3751-ROCLIS, 3752-ROCLIS, 3753-ROCLIS, 3754-ROCLIS, 3755-ROCLIS, 3756-ROCLIS, 3765-ROCLIS, 3766-ROCLIS, 3767-ROCLIS, 3768-ROCLIS, 3769-ROCLIS, 3836-ROCLIS, 3838-ROCLIS, 3840-ROCLIS, 3841-ROCLIS, 3842-ROCLIS, 3854-ROCLIS, 3855-ROCLIS, 3856-ROCLIS, 3857-ROCLIS, 3858-ROCLIS, 3859-ROCLIS, 3860-ROCLIS, 3861-ROCLIS, 3862-ROCLIS, 3863-ROCLIS, 3864-ROCLIS, 3865-ROCLIS, 3866-ROCLIS, 3867-ROCLIS, 3869-ROCLIS, 3870-ROCLIS, 3871-ROCLIS, 3872-ROCLIS, 3873-ROCLIS, 3BS, 4103-ROCLIS, 4163-ROCLIS, 5089-ROCLIS, 5092-ROCLIS, 5427-ROCLIS, 5475-ROCLIS, 5485-ROCLIS, 5543-ROCLIS, 56027-ROCLIS, 6594-ROCLIS, 6595-ROCLIS, 6597-ROCLIS, 7449-ROCLIS, 7749-ROCLIS, 7750-ROCLIS, 7752-ROCLIS, 82028-ROCLIS, 8343-ROCLIS, 8368-ROCLIS, 8412-ROCLIS, 8476-ROCLIS, 87921-ROCLIS, 89115-ROCLIS, 89847-ROCLIS, 9158-ROCLIS, EAG, GLU, GLUDKA, GLUNOTE, GLUOB, GLUT2, GLUWB, UGLU24, 1017870401, 115921075, 1162938491, 120390231, 127135259, 131441048, 144542999, 1957880593, 2115830499, 2203526069, 246226825, 251010311, 27174643, 27174693, 27175434, 27176768, 27178073, 27178197, 27178427, 27178448, 289393779, 3006516, 30261332, 30261362, 30983435, 30983437, 30983449, 31204734, 326179445, 35622651, 39939013, 40019019, 4422110, 4422111, 4422112, 4422113, 4422114, 4422115, 4422116, 4422118, 4425996, 4426000, 45762262, 4629103, 4629104, 4629108, 4629109, 4629110, 7403375, 7403376, 7403469, 7403500, 7403501, 7441097, 819030294, 819030916, 840182806, 840846518, 1HROB, 2HPP, BS1HR, BS1OB, BS2HR, BS2OB, BS3HR, BS3OB, BS4HR, FAST, FASTB, FBS, G6PD, GLU-B, GLUC, GLUSF, HGLUC, MGLBF, PGLUC, UGLU, UGLU1, UGLU2, UGLU3, UGLUF |
| Dual x-ray absorptiometry | 76977, 77078, 77079, 77080, 77081, 77082, 77083, 77085, 77086, 78350, 78351, 81405, 81406, 0508T, 0554T, 0555T, 0556T, 0557T, 0558T, G0062, G0063, G0130, G0131, G0132, G0133, G8106, G8107, G9769 |
| Influenza vaccine | 90657, 90658, 90660, 90655, 90656, 90661, 90662, 90664, 90666, 90667, 90668, Q2035, Q2036, Q2037, Q2038, Q2039, 90654, G0919, Q2034, 90653, 90672, 90685, 90686, 90687, 90688, Q2033, 90673, 90630, 90674, 90682, 90756, 90689, 90694 |
| Pneumococcal vaccine | 90669, 90670, 90671, 90677, 90732, G0009, S0195 |
| Herpes zoster vaccine | 90736, 90750 |

**Supplemental table 4.** Clinical manifestations and organ involvement of patients with systemic lupus erythematosus (SLE) from the Lupus Midwest Network cohort at or ever prior to January 1, 2015.

|  | **N=440 (%)** |
| --- | --- |
| **SLE duration**, median (IQR) | 10.7 (4.6-20.3) |
| **Constitutional** |  |
| Fever | 26 (5.9) |
| **Hematologic domain** |  |
| Leukopenia | 187 (42.5) |
| Thrombocytopenia | 67 (15.2) |
| Autoimmune hemolysis | 15 (3.4) |
| **Neuropsychiatric domain** |  |
| Delirium | 2 (0.5) |
| Psychosis | 1 (0.2) |
| Seizure | 6 (1.4) |
| **Mucocutaneous domain** |  |
| Non-scarring alopecia | 20 (4.5) |
| Oral ulcers | 37 (8.4) |
| Subacute cutaneous or discoid lupus | 75 (17.0) |
| Acute pericarditis | 109 (24.8) |
| **Serosal domain** |  |
| Pleural or pericardial effusion | 76 (17.3) |
| Acute pericarditis | 39 (8.9) |
| **Musculoskeletal domain** |  |
| Inflammatory arthritis | 286 (65.0) |
| **Renal domain** |  |
| Proteinuria >0.5g/24hrs | 103 (23.4) |
| Class II or V lupus nephritis | 42 (9.5) |
| Class III or IV lupus nephritis | 71 (16.1) |
| **Immunology domain** |  |
| Antiphospholipid antibodies | 114 (25.9) |
| Low C3 or low C4 | 158 (35.9) |
| Low C3 and low C4 | 147 (33.4) |
| Anti-dsDNA | 327 (74.3) |
| Anti-Smith | 93 (21.1) |
| **DMARD treatment** | 373 (84.8) |

DMARD: Disease modifying anti-rheumatic drug; IQR: Interquartile range.

**Supplemental table 5.** Number of patients with and without systemic lupus erythematosus (SLE) at risk at each timepoint during the assessment of preventive services in the Lupus Midwest Network cohort between 2015 and 2020.

|  | **Number of patients at risk** | | | | | | | |
| --- | --- | --- | --- | --- | --- | --- | --- | --- |
| **Preventive services** | **Baseline** | | **1 year** | | **3 years** | | **5 years** | |
|  | **SLE** | **Non-SLE** | **SLE** | **Non-SLE** | **SLE** | **Non-SLE** | **SLE** | **Non-SLE** |
| **Breast cancer screening** | 164 | 163 | 69 | 66 | 24 | 28 | 13 | 10 |
| **Cervical cancer screening** | 257 | 256 | 206 | 198 | 141 | 120 | 89 | 61 |
| **Hypertension screening** | 219 | 279 | 41 | 77 | 9 | 22 | 4 | 4 |
| **Hyperlipidemia screening** | 318 | 309 | 222 | 220 | 107 | 130 | 61 | 58 |
| **Diabetes mellitus screening** | 398 | 390 | 63 | 182 | 15 | 80 | 7 | 30 |
| **Osteoporosis screening** | 371 | 411 | 316 | 390 | 243 | 339 | 178 | 264 |
| Age ≥65 years old | 86 | 98 | 67 | 91 | 44 | 69 | 30 | 52 |
| Age <65 years old | 285 | 313 | 249 | 299 | 199 | 270 | 148 | 212 |
| Glucocorticoid use* |  |  |  |  |  |  |  |  |
| ≥90 days | 230 | 26 | 195 | 25 | 147 | 21 | 108 | 15 |
| <90 days | 141 | 385 | 121 | 365 | 96 | 318 | 70 | 249 |

*At index date (January 1, 2015).

**Supplemental table 6.** Number of patients with and without systemic lupus erythematosus (SLE) at risk at each timepoint during the assessment of immunizations in the Lupus Midwest Network cohort between 2015 and 2020.

|  | **Number of patients at risk** | | | | | | | | | |
| --- | --- | --- | --- | --- | --- | --- | --- | --- | --- | --- |
| **Immunization** | **Baseline** | | **1 year** | | **2 years** | | **3 years** | | **5 years** | |
|  | **SLE** | **Non-SLE** | **SLE** | **Non-SLE** | **SLE** | **Non-SLE** | **SLE** | **Non-SLE** | **SLE** | **Non-SLE** |
| Influenza | 439 | 430 | 162 | 199 | 115 | 156 | 88 | 130 | 66 | 76 |
| Pneumococcal disease | 146 | 271 | 134 | 251 | 119 | 230 | 94 | 209 | 70 | 154 |
| Herpes zoster* | 220 | 231 | 183 | 201 | 137 | 150 | - | - | - | - |

*After recombinant zoster vaccine became available (January 1, 2018).
